# Expression Analysis of Circular RNAs in Young and Sexually Mature Boar Testes

**DOI:** 10.3390/ani11051430

**Published:** 2021-05-17

**Authors:** Fei Zhang, Xiaodong Zhang, Wei Ning, Xiangdong Zhang, Zhenyuan Ru, Shiqi Wang, Mei Sheng, Junrui Zhang, Xueying Zhang, Haiqin Luo, Xin Wang, Zubing Cao, Yunhai Zhang

**Affiliations:** 1 Anhui Province Key Laboratory of Local Livestock and Poultry, Genetical Resource Conservation and Breeding, College of Animal Science and Technology, Anhui Agricultural University, Hefei 230036, China; zhfei@ahau.edu.cn (F.Z.); xdzhang1983@ahau.edu.cn (X.Z.); 18856899296@163.com (W.N.); xiangdongzhang2021@163.com (X.Z.); Ruzhenyuan@163.com (Z.R.); wsq1013491116@163.com (S.W.); sm15955268515@163.com (M.S.); zjr2432985486@163.com (J.Z.); zhangxueying1203@163.com (X.Z.); wssxl0513@163.com (H.L.); wx3465751162@163.com (X.W.); 2 School of Life Sciences, Anhui Agricultural University, Hefei 230036, China

**Keywords:** circular RNAs, pig, testis, sexual maturity, expression profile

## Abstract

**Simple Summary:**

Circular RNAs are novel long non-coding RNA involved in the regulation of gene expression. Recently, the expression of circRNAs was characterized in testes of humans and bulls. However, the profiling of circRNAs and their potential biological functions in boar testicular development are yet to be known. In this study we characterized expression and biological roles of circRNAs in piglet (30 d) and adult (210 d) boar testes by high-throughput sequencing. We identified a large number of circRNAs during testicular development, of which 2326 circRNAs exhibited a significantly differential expression. Gene ontology analysis revealed that these differential expressed circRNAs might be involved in regulating spermatogenesis and hormone biosynthesis. Overall, the results indicate that circRNAs are abundantly expressed in boar testes and exhibit dynamic changes during testicular development. These findings will enable the provision of potential molecular markers for both breeding of elite boars and evaluating developmental status of boar testes.

**Abstract:**

Testicular development is critical for male animals’ reproduction and is tightly regulated by epigenetic factors. Circular RNAs (circRNAs) were recently identified in the testes of humans and bulls. However, the expression profile of circRNAs and their potential biological functions in boar testicular development remain unclear. We identified 34,521 and 31,803 circRNAs in piglet (30 d) and adult (210 d) boar testes by high-throughput sequencing, respectively. Bioinformatics analysis revealed that these circRNAs are widely distributed on autosomes and sex chromosomes. Some of the host genes can generate multiple circRNAs. A total of 2326 differentially expressed circRNAs (DECs) derived from 1526 host genes was found in testicular development, of which 1003 circRNAs were up-regulated in adult boar testes and 1323 circRNAs were down-regulated. Furthermore, gene ontology analysis of host genes of DECs revealed that these circRNAs are mainly involved in regulating spermatogenesis, cilia motility, and hormone biosynthesis. The Kyoto Encyclopedia of Genes and Genomes pathway enrichment analysis revealed that the DECs are markedly enriched to stem cell pluripotency regulation, tight junctions, adhesion junctions, and cAMP signaling pathway. These results indicate that circRNAs are abundantly expressed in boar testes and exhibit dynamic changes during testicular development.

## 1. Introduction

The domestic pig not only provides enriched animal derived food products for humans, but also is increasingly used as an animal model for human disease in biomedicine, while the boar fertility in the swine industry determines the productive efficiency of commercial pork and the progress of genetics in the pig-breeding program. It should be noted that the developmental status of boar testes frequently affects the sperm quality, which in turn determines the boar fertility [1]. Accumulating evidence revealed that the testicular development in pigs is tightly regulated by multiple genetic factors [2]. However, the epigenetic mechanisms underlying boar testicular development remain largely unclear.

Testis is the main reproductive organ of male animals, the functions of which are spermatogenesis and androgen secretion. Androgens are produced by testicular mesenchymal cells and play significant roles in maintaining spermatogenesis and accessory sexual gland development. Thus, spermatogenesis and androgen secretion both affect the physiological reproductive processes of male animals [3]. The development of testis starts from the formation of testicular primordium in the embryonic stage. Leydig and Sertoli cells begin to proliferate and differentiate. From postnatal to prepuberty, the development of spermatogenic cells is in a state of stagnation, and the convoluted seminiferous tubules are almost parenchymal without obvious lumen. Mammalian testicular development and spermatogenesis are highly precise and orderly processes with clearly distinguished stages. Accordingly, testicle-specific genes are expressed in strict accordance with temporal and spatial factors at different developmental stages and participate in the regulation of many aspects of sexual development and function under the action of numerous different factors [4,5]. The normal development of testes requires the regulation of cell-cycle-related genes such as cyclin and cyclin-dependent kinase (*CDK*) [6] as well as meiosis-related genes such as deleted in azoospermia (*DAZ*) family genes [7]; stimulated by retinoic acid gene 8 (*STRA8*) [8]; synaptonemal complex protein gene; heat shock protein gene (*HSP*); proto-oncogene; spermatogenesis related genes such as transition nuclear protein genes (*TNP*), protamine gene 1(*PRM1*) [9], tektin gene (*TEKTIN*), cation channel of sperm (CatSper) family genes, and transcription regulators; and apoptosis related genes such as *Bax*/*Bcl-2* (B cell lymphoma-2) family genes, *P53* gene [10], *Fas*/FasL (Fas Ligand) genes, and *c-kit* (c kitproto oncogene)/SCF (Stem cell factor) genes.

The extensive application of molecular biology technology has made it possible to elucidate the molecular regulatory mechanisms in genomes, and it has been increasingly applied to the field of animal breeding and reproduction. Gene regulation is the fundamental cause of all biological behaviors and phenotypes. It is a multi-level, multi-channel, multi-mode spatiotemporal dynamic process that mainly occurs at the transcription and post-transcription levels. In a narrow sense, the term “transcriptome” typically refers to the total mRNA of an organism. In a broader sense, it refers to all the RNAs, including mRNA and non-coding RNA (ncRNA), such as microRNA (miRNA), circular RNA (circRNA), long non-coding RNA (LncRNA), small interfering RNA (siRNA), and PIWI-interacting RNA (piRNA) transcribed by an organism in a specific environment or physiological state. Research into ncRNA has become a scientific frontier in the post-genomic era and accounts for an important area of functional genomic research. Researchers have studied the effects of many different types of genetic profiles, such as gene expression [11], proteome [12], small RNA [13], and piRNA [14] profiles on testicular development and/or spermatogenesis in mice.

Epigenetics is a new branch of genetics, which has become one of the most popular research fields in life science, including DNA methylation, histone modification and ncRNA, chromatin remodeling, and genomic imprinting. Proper regulation of DNA methylation is necessary for normal testicular development and maturation [15]. Spermatogenesis depends on precise histone modification regulation. Knockout of histone H3K9 deacetylase Jmjd1a in mice leads to the increase of histone acetylation level in sperm cells and germ cell apoptosis, which leads to micro-testis, oligospermia, and infertility [16]. NcRNAs are divided into short non-coding RNA (small RNA) and LncRNA according to the length of transcripts [17]. These ncRNAs mainly exert a critical role in spermatogenesis. However, the role of ncRNA in testicular development remains largely unclear.

CircRNAs are a recently discovered new class of ncRNA and generated by pre-mRNA back splicing. Unlike linear RNA, CircRNA forms a covalently closed circular structure and has shown great application potential as a gene regulatory factor [18] involved in a variety of biological processes such as growth, development, reproduction, and disease. CircRNAs can be divided into three categories according to their sequence construction and the location of the source genome, i.e., exon circRNAs, intron circRNAs (ciRNAs), and exon-intron circRNAs (EIciRNAs). Recently, circRNAs have been proved to be rich in conserved miRNA response elements (MREs), and act as competing endogenous RNAs (ceRNAs) or natural miRNA sponges in regulating gene expression, they communicate with and co-regulate each other by competing for binding to shared miRNA [19]. Exon circRNAs are mainly found in the cytoplasm and act as an miRNA sponge [20], while ciRNAs and EIciRNAs are thought to be regulators of parental gene in the cis/transcribed nucleus [21]. Some circRNAs can also regulate gene expression by competitive alternative splicing or by acting as a translation template [22]. Another promising application for circRNA research is the development of novel biomarkers for diseases [23]. It has been reported that circRNAs are abundant in the testes or semen supernatants of bulls [24], humans [25], and boars [26]. Meanwhile circRNAs were also identified in porcine sperm and related to sperm motility [27]. Deep sequencing of the transcriptomes of multiple tissues and organs including the brain and testis in rats has demonstrated that the level of tissue-specific circRNAs in the testis is second only to that of brain tissue [28]. It has been shown that the pig circRNAs show modest sequence conservation with human and mouse circRNAs, are flanked by long introns, exhibit low abundance, and are expressed dynamically in a spatiotemporal specific manner [26]. Though they revealed circRNA expression landscape in testes of boars, Liang et al. did not provide differently expressed circRNAs of testis between prepubertal young and sexually matured adult boars. The function of testicular-specific circRNA was initially reported to be that of a miR-138 sponge [20], indicating that circRNAs may be involved in the complex regulatory network of testicular development and spermatogenesis. CircRNAs are then thought to be involved in mammalian reproduction regulation and related to sperm motility and activity [25]. Therefore, studying changes in the circRNA expression profile of boar testes upon sexual maturation can reveal the characteristics of the transcriptome and the regulatory molecular mechanisms of boar testicular development.

In this study, we performed the circRNA expression profiles of young and sexually mature Landrace boar testicles. This study laid a foundation for further study on the relationship between circRNAs and testicular function and reproductive regulation in breeding pigs.

## 2. Materials and Methods

### 2.1. Sample Collection

The testicles of young (30-day-old, three boars, average weight 8.5 kg, labelled Piglet-D30) and sexually mature (210-day-old, three boars, average weight 155 kg, labelled Adult-D210) Landrace boars were obtained from Anhui Hoshine Agro-Pastoral Co., Ltd., Anhui, China. For the sexually mature group, adult boars were undergoing normal spermatogenesis as evidenced by seminal parameters prior to harvesting testis for experiment. One of the testes was randomly selected for RNA isolation and histological analyses. The testicular samples were immediately cut into small pieces, transferred into cryogenic vials, and stored in liquid nitrogen. Samples were transported to LC Sciences, Hangzhou, China for subsequent library construction and sequencing.

### 2.2. Morphological and Histological Assessment

The testicles were cut into small pieces, washed in precooled DPBS three times, and then fixed in Bouin’s fixative solution. Paraffin embedded tissues were used to make histological sections. Following hematoxylin and eosin (H & E) staining, the images were acquired using microscopy (Olympus DP72).

### 2.3. Total RNA Isolation, Purification, and Quality Control

Total RNA was isolated and purified from each sample using TRIzol reagent (Invitrogen, Carlsbad, CA, USA) according to the manufacturer’s protocol. Then, NanoDrop ND-1000 (NanoDrop, Wilmington, DE, USA) was used to determine the amount and purity of total RNA, and Agilent 2100 was used to test the integrity of the RNA with an RNA integrity number > 7.0 being taken as the standard of total RNA quality.

### 2.4. cDNA Library Construction and Sequencing

Aliquots of total RNA (5 μg) from each group were taken and the ribosomal RNA was removed using a Ribo-ZeroTM rRNA Removal Kit (Illumina, San Diego, CA, USA) according to the manufacturer’s instructions [29]. The left RNA was then incubated at 37 °C for 30 min with 5 U/μg RNase R (Epicentre Inc, Madison, WI, USA) to remove linear RNA. After removing ribosomal and linear RNAs, the enriched circRNAs were fragmented into small pieces using divalent cations under high temperature conditions. Then the cleaved RNA fragments were reverse-transcribed to produce the cDNA, which was next used to synthesize U-labeled second-stranded DNAs using TruSeq Stranded Total RNA HT Sample Prep Kit (Illumina, San Diego, CA, USA) with RNase H, E. coli DNA polymerase I and dUTP. An A-base was then added to the blunt ends of each strand, preparing them for ligation to the indexed adapters. Each adapter contained a T-base overhang for ligating the adapter to the A-tailed fragmented DNA. Single- or dual- index adapters were ligated to the fragments, and size selection was performed with AMpure XP beads. After the heat-labile UDG enzyme treatment of the U-labeled second-stranded DNAs, the ligation product was amplified by PCR under the following conditions: Initial denaturation at 95 °C for 3 min, denaturation at 98 °C for 15 s, annealing at 60 °C for 15 s, extension at 72 °C for 30 s, a total of 8 cycles, finally extending for 5 min at 72 °C. Six cDNA libraries with an average fragment size of 300 bp (±50 bp) were generated. Finally, 150 bp paired-end sequencing was performed on an Illumina Hiseq4000 (LC Sciences, Hangzhou, China) according to the supplier’s recommended protocol.

### 2.5. Sequence Map and circRNA Prediction

The Cutadapt (V1.10) software [30] was used to remove the reads containing adaptor contamination and low-quality bases and undetermined bases. i.e., those with more than 50% low-quality (Q Value ≤ 20) bases or more than 10% unknown nucleotides (N), to obtain clean reads. For further analyses, the raw sequencing data was filtered to obtain clean data (Table S1). FastQC software version V0.10.1 (http://www.bioinformatics.babraham.ac.uk/projects/fastqc/, accessed on 3 May 2012) was then used to validate the quality of sequences. TopHat2 (V.2.0.4, mate-inner-dist 50, mate-std-dev 20, min-anchor 4, min-intron-length 20, max-intron-length 500,000, read-edit-dist 2, min-segment-intron 70, max-segment-intron 40,000, read-mismatches 2, library-type fr-firststrand) [31] was used to map the clean reads to the reference genome (Sus_scrofa V88). Remaining reads (unmapped reads) were still mapped to the genome using TopHat-Fusion software version V2.1.0 (Center for Bioinformatics and Computational Biology, MD, USA) [32]. CIRCExlorer2 software version V2.2.6 (Key Laboratory of Computational Biology, Shanghai, China) [33] was used to de novo assemble the mapped reads to circular RNAs at first; Then, back splicing reads were identified in unmapped reads by TopHat-Fusion software version V2.1.0 and CIRCExplorer2 software version V2.2.6. Statistical analysis was performed on the identified circRNAs in terms of type, chromosome distribution, and length distribution. The length of circRNAs was indeed calculated based on the exonic regions. circRNA was assembled based on the assumption that introns and intergenic regions are spliced, and the length of circRNA calculated based on the assembled sequence.

### 2.6. Expression Profile and Analysis of Differentially Expressed circRNAs

The total number of reads spanning back-spliced junctions was served as an absolute measure of circRNA abundance. SRPBM (number of circular reads/number of mapped reads (units in billion)/read length) reads were denoted to estimate the relative expression of a circRNA [34]. Raw counts were used as input for edgeR [35] to identify differentially expressed circRNAs across samples and groups. The differential expression of circRNAs before and after sexual maturity was determined by log2|fold_change| ≥ 1 and features with FDR-adjusted *p* Value less than 0.05.

To identify the possible functions of differentially expressed circRNAs and their host genes involvement in the common biological processes, we selected host genes of differentially expressed circRNAs for Gene Ontology (GO) analysis and Kyoto Encyclopedia of Genes and Genomes (KEGG) analysis. The host genes of differentially expressed circRNAs were classified into three categories of the GO database: biological processes, cellular components, and molecular functions. The KEGG database was used to ascribe identified host genes of differentially expressed circRNAs to biological mechanisms and cellular pathways. GO and KEGG enrichment analysis was performed using (http://geneontology.org, accessed on 2 July 2018 and http://www.kegg.jp/kegg, accessed on 1 January 2018).

### 2.7. miRNA Sponge Analyses

The interaction between circRNA and miRNA was analyzed by Targetscan software version V5.0 (Whitehead Institute for Biomedical Research, MA, USA) and miRanda software version V3.3a (Memorial Sloan-Kettering, NY, USA). Targetscan predicted miRNA target based on seed region [36]. miRanda tool was used to predict the potential miRNA binding sites on the circRNA [37]. The filter thresholds are Targetscan-Score ≥ 50 and miRanda-Energy < −10.

### 2.8. Validation of circRNA Using Quantitative Reverse Transcription PCR

In order to verify the accuracy of the circRNA data obtained by sequencing, total RNA was incubated at 37 °C with 5 U/μg of RNase R (Epicenter Bio-technologies, Mumbai suburban, India). RNA without RNase R treatment served as control group. Reverse transcription was performed using a Quanti Tect Reverse Transcription Kit (Qiagen, Hilden, Germany according to the manufacturer’s instructions. Quantitative Reverse Transcription PCR (RT-qPCR) was performed on a StepOne Plus real-time PCR system (Applied Biosystems, CA, USA) using SYBR Green PCR Master Mix (Roche, Basel, Switzerland). The reaction conditions were as follows: 1 cycle at 95 °C for 2 min and 5 s at 95 °C, 10 s at 60 °C, 40 cycles. Relative expression and 2(-Delta Delta C(T)) method were used to quantitatively analyze gene expression [38]. The expression levels of circRNAs were calculated after normalization to *EF1α1* levels in each sample. The primers used are listed in Table S2. Each experiment was performed in three independent replicates.

### 2.9. Statistical Analysis

RT-qPCR was repeated three times. Differences between the data were analyzed by SPSS (V25.0) using a Student’s *t*-test. Values were expressed as mean ± standard error of the mean (mean ± S.E.M). *p* value < 0.05 was considered statistically significant.

## 3. Results

### 3.1. Morphological and Histological Characteristics of Testicular Tissue

**Figure 1:**
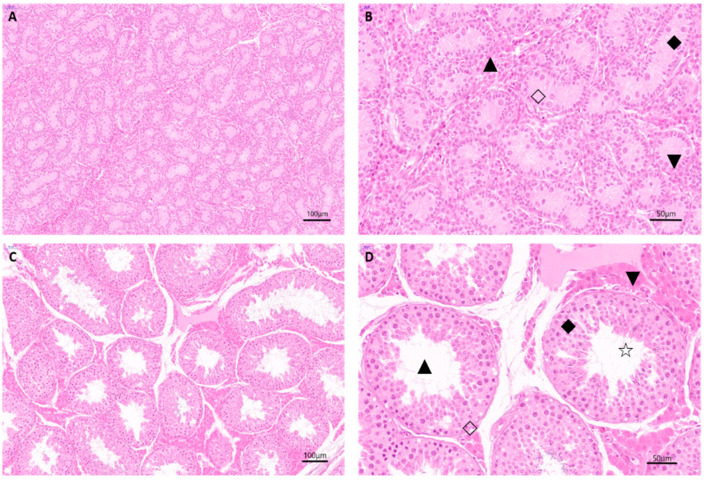
Morphological and histological analysis of the testes tissues of young (Piglet-D30) and sexually mature (Adult-D210) boars. Different magnifications of young boar testis tissues (**A**,**B**). The seminiferous tubule structure is formed, the lumina are in an atresia state, and it contains only one type of germ cell, namely the spermatogonia. Bars = 100 μm and 50 μm. Different magnifications of mature boar testis tissues (**C**,**D**). The seminiferous tubules have clear lumina and contain germ cells at different periods. Bars = 100 μm and 50 μm. ▲ Seminiferous tubule; ▼ interstitial cells; ◆ spermatogonia cells; ◇ sustentacular cells; ☆ sperm.

Testis tissue staining showed that there are many types of cells in the testicular tissues of young Landrace breeding boars, such as Leydig cells, spermatids, and Sertoli cells (Figure 1A,B). A seminiferous tubule structure was formed and was thin and tightly arranged, but the lumen was still in a locked state. Spermatogonium are the only type of germ cells present in the testicular tissues of the young boars, which lack spermatocytes and spermatozoa. Testicular interstitial cells are uniformly distributed around seminiferous tubules, and spermatogonia are neatly arranged along the seminiferous tubule basement membranes.

Conversely, the seminiferous tubules in the testicular tissues of sexually mature Landrace boars are clear and complete, with lumina clearly observed in the center of the seminiferous fine tube cords, forming seminiferous tubules (Figure 1C,D). There are multiple layers of cells in lumina, and mature sperm are observed. These results indicate that spermatogenesis is present in the seminiferous tubules of the sexually mature boars.

### 3.2. Basic Characteristics of circRNA

**Figure 2:**
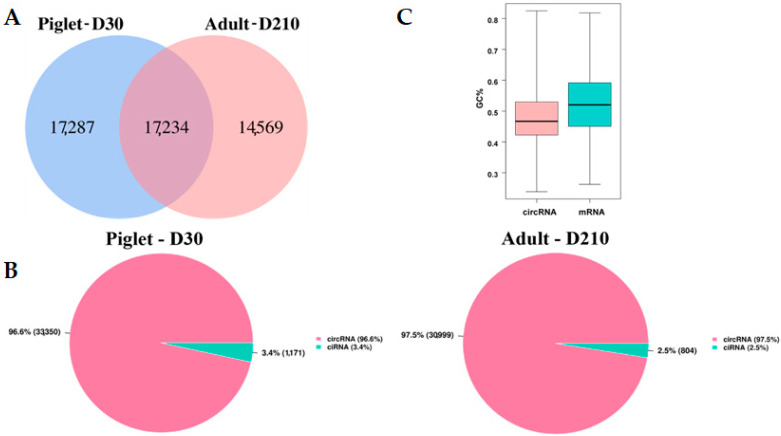
Basic characteristics of circRNAs in the testes of young (Piglet-D30) and sexually mature (Adult-D210) boars. Venn diagram showing the amount of circRNA expression in the testes tissues of young and sexually mature Landrace boars (**A**). Distribution of circRNA types in the testis tissue of young and sexually mature Landrace breeding boars. All circRNAs are divided into two types: exon circRNA, and intron circRNA (ciRNA) (**B**). GC contents of circRNA obtained from sequencing compared with the GC contents of mRNA in the database (**C**).

To investigate the expression profile and possible biological functions of circRNAs in boar testicular tissues before and after sexual maturity, this study used total RNA-Seq (with removal of ribosomal and linear RNAs) to sequence and analyze the circRNAs in the testicular tissues of young and sexually mature Landrace boars. A total of 49,090 candidate circRNAs were obtained from the testis before and after sexual maturity (Table S3). Among them, 34,521 and 31,803 circRNAs were detected in the Piglet-D30 and Adult-D210 samples, respectively, with 17,287 unique to the Piglet-D30 group, 14,569 unique to the Adult-D210 group, and 17,234 circRNAs in both groups (Figure 2A). Using CIRCExlorer2 software, circRNAs can be divided into exon-circRNA, and intron-circRNA according to their position on the genome. Here, the Piglet-D30 group contains 96.6%, and 3.4% of these two types, respectively, while the Adult-D210 group contains 97.5%, and 2.5%, respectively (Figure 2B). The GC content of all the circRNAs obtained by sequencing is 48.02% and that of their mRNA is 52.11% (Figure 2C).

**Figure 3:**
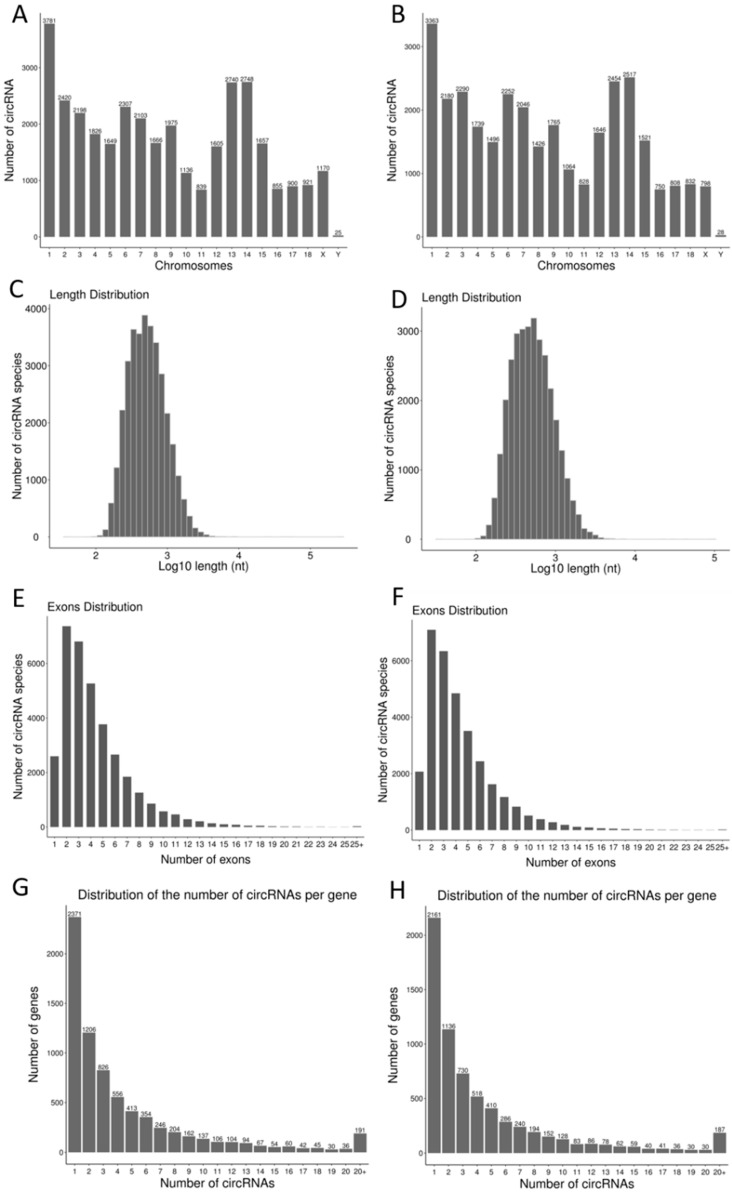
Comparison of basic characteristics of circRNA in testes tissues of young (Piglet-D30) and sexually mature (Adult-D210) boars. Distribution of circRNAs on chromosomes in testis tissues of young and sexually mature Landrace breeding boars (**A**,**B**). Length distributions of circRNA in testis tissues of young and sexually mature Landrace breeding boars (**C**,**D**). Analysis of the number of exons contained in exon circRNA in the testis tissues of young and sexually mature Landrace breeding boars (**E**,**F**). Number of circRNAs produced by genes of different origin in the testis tissues of young and sexually mature Landrace breeding boars (**G**,**H**).

The 49,090 circRNAs are widely distributed on all chromosomes (Figure 3A,B). The shortest sequence circRNA is 98 nt in length while the longest is 496,661 nt, with most (28,947, 58.97%) have lengths in the range 100–10,000 nt (Figure 3C,D). The sequenced circRNAs consist of at least one exon and a maximum of 65 exons, with most (42,485, 86.54%) consisting of no more than seven exons and 2597 circRNAs in the Piglet-D30 group consisting of only one exon (Figure 3E). In the Adult-D210 group, 3066 circRNAs consist of only one exon (Figure 3F). The results show that one source gene may generate several different circRNAs, with 49,090 circRNAs being derived from 8254 source genes. Approximately 500 circRNAs out of the 40,000 circRNAs are >10 RPM (Reads of exon model per million mapped reads), in general the potential circRNAs were found at low abundance. For Piglet-D30, the circRNAs are derived from 7304 genes, and 2371 source genes generate only one circRNA (Figure 3G). There are 6687 source genes for Adult-D210, 2161 of which they only produce one circRNA (Figure 3H).

### 3.3. Identification of Differentially Expressed circRNAs in Testicular Tissues of Young and Sexually Mature Landrace Boars

**Figure 4:**
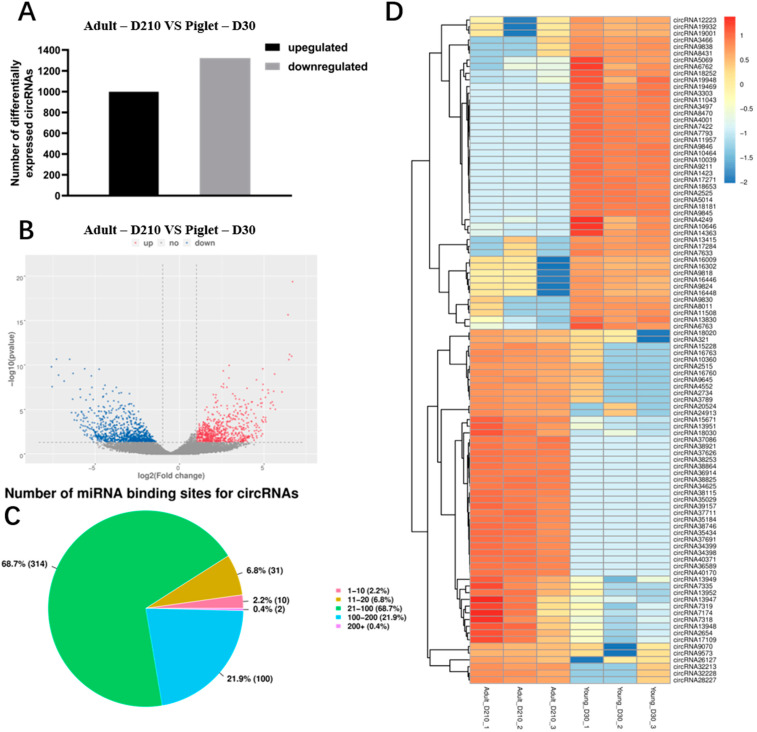
Differentially expressed circRNAs in testes tissues of young (Piglet-D30) and sexually mature (Adult-D210) boars. Significantly differently expressed circRNAs in the testis tissues of young and sexually mature Landrace breeding boars, with upregulated circRNAs on the left and downregulated circRNAs on the right. *p* Value < 0.05, and log2 (fold change) > 1 or log2 (fold change) < −1 (**A**). Volcano plot of significantly differently expressed circRNA, where red represents the upregulated circRNAs and blue represents the downregulated circRNAs (**B**). Analysis of the proportion of circRNA processing different numbers of miRNA targets (**C**). Heat map showing the changes of the first 100 significantly differently expressed circRNAs in the testis tissues of young and sexually mature Landrace breeding boars. Red represents upregulation and blue represents downregulation (**D**).

The circRNA expression profiles of the testicles of the young and sexually mature Landrace boars were analyzed to identify the circRNAs that are suggestive differentially expressed. The experimental results were analyzed in terms of the RPM model to quantify circRNA abundance and to calculate the significance of differences between groups. A total of 2326 differentially expressed circRNAs (*p* Value < 0.05) were identified between the Piglet-D30 and Adult-D210 groups (Table S4). Among the Adult-D210 group, 1003 circRNAs were significantly upregulated and 1323 circRNAs were significantly downregulated compared to the Piglet-D30 group (Figure 4A,B). One of the most important biological functions of circRNA is to act as a highly efficient competitive endogenous RNA, which can adsorb miRNA, thus regulating expression of its target gene. Furthermore, each circRNA may combine with one or more target miRNAs. Targetscan and miRanda were used to predict the putative miRNA targets on circRNAs. Further statistical analysis of circRNA binding sites on 457 miRNAs found that most (447, 97.8%) miRNAs have at least 10 circRNA binding sites, of which the proportion of miRNAs containing 21–100 circRNA binding sites is the highest (Figure 4C), suggesting that circRNAs in the testes may regulate testicular development and spermatogenesis by actively participating in binding to miRNAs.

Hierarchical clustering analysis of the first 100 circRNAs based on *p* Value that are differentially expressed in the two groups was performed. As shown in the heat map (Figure 4D), samples at the same stage were clustered together, with red and blue representing significant (*p* Value < 0.05) increases or decreases, respectively, in the Adult-D210 group as compared to the Piglet-D30 group. The results show that circRNAs exhibit a clear differential expression during testicular development, and that the circRNAs involved in gene expression regulation (circRNA 12223, derived from *UMODL1* gene), histone acetylation (circRNA 5069, derived from *CREBBP* gene), motor behavior (circRNA 8431, derived from *APBA2* gene), small GTPase-mediated signal transduction (circRNA 18252, derived from *PLCE1* gene) and extracellular exosomes (circRNA 3303, derived from *CPNE8* gene) are significantly upregulated (*p* Value < 0.05).

### 3.4. GO and KEGG Signal Pathway Enrichment Analyses

CircRNA are usually produced by exon regions of coding genes through reverse splicing events [18]. In part, the function of circRNA may be inferred by its source gene [39]. In this study, GO and KEGG signal pathway enrichment analyses were performed on the generated source genes to predict their possible functions during testicular development and sperm maturation. GO enrichment analysis can provide insight into the functions of circRNAs.

**Figure 5:**
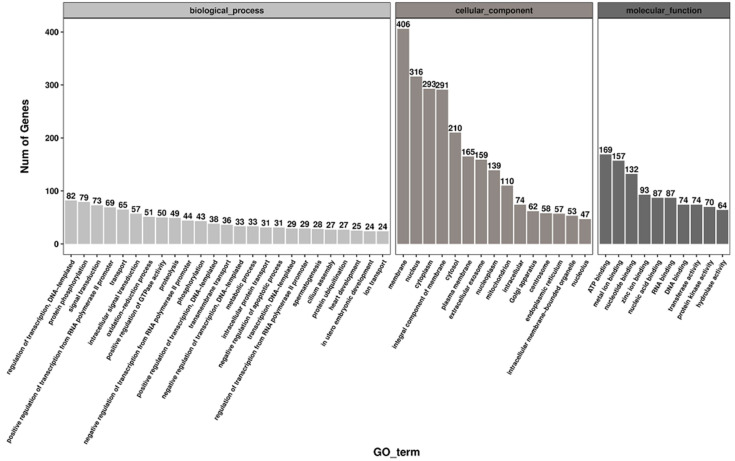
Gene ontology (GO) analysis of differentially expressed circRNA source genes. GO analysis of the top enriched terms of the differentially expressed circRNA hosting genes identified in young (Piglet-D30) and sexually mature (Adult-D210) boar testes. The source genes are divided into three ontologies by GO analysis (left: biological process, middle: cells, organ components, right: molecular function).

According to the GO database, the source genes for suggestive differentially expressed circRNAs in the two age groups are divided into three ontologies: biological processes, cellular components, and molecular function. The 1527 source genes of 2326 significantly differently expressed circRNAs (*p* Value < 0.05) obtained from the sequencing results are completely enriched in 4716 GO items (Table S5). Among them, 3069 GO items are related to biological processes, 166 of which have significant differences (*p* Value < 0.05), including smoothened signaling pathways, piRNA metabolism processes, spermatogenesis, flagellar assembly, and stem cell development. A total of 580 GO items are related to the cellular location of genes, of which 33 are significantly different (*p* Value < 0.05), including centrosome, flagella, cell connections, and sex chromosomes. Furthermore, 961 GO items are related to the molecular functions of genes, of which 444 items are significantly different, (*p* Value < 0.05), including phospholipid transport ATPase activity, transcription activator activity, and phosphatase binding. Figure 5 shows the number of differential genes for the biological process, cell components, and molecular functions, GO terms in descending order, with the top 25, 15, and 10 GO items, respectively.

**Figure 6:**
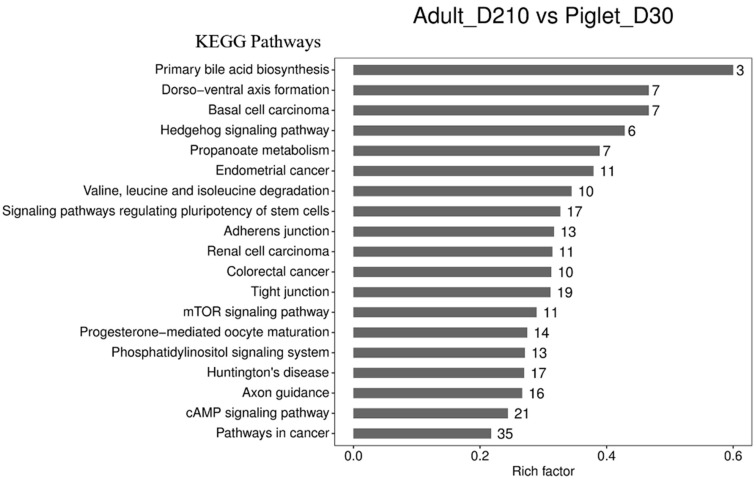
Kyoto Encyclopedia of Genes and Genomes (KEGG) analysis of hosting genes of differentially expressed circRNAs. Analysis of KEGG signals pathways of the source genes of circRNAs with the largest differential expression in young (Piglet-D30) and sexual mature (Adult-D210) boar testes. All 19 KEGG signal pathways with significant differences are listed.

Moreover, analysis of the results showed that 1527 source genes that produce significant differences in circRNA expression (*p* Value < 0.05)during testicular development are enriched in 249 signal pathways (Table S6), 19 of which are significantly different (*p* Value < 0.05) (Figure 6), such as signal pathways that regulate stem cell pluripotency (*AKT3*, *AVCVR2A*, *FGFR1*, *ACVR1*, *FZD3*, *SMAD4*, etc.), tight junction (*MYH15*, *PRKCA*, *AMOTL1*, *PPP2CB*, *CDC42*, *PTEN*, etc.) and adhesion connections (*LEF1*, *NECTIN3*, *SMAD2*, *AFDN*, etc.), hedgehog signaling pathways (*HHIP*, *GSK3B*, *PTCH1*, *SMO*, *RAB23*, etc.), cAMP signaling pathways (*PLD1*, *TIAM1*, *GNAI1*, *ADCY1*, etc.), mTOR signaling pathways (*BRAF*, *RICTOR*, *HIF1A*, etc.), and phosphatidylinositol signaling systems (*CDS1*, *DGKH*, *PLCB1*, *DGKK*, etc.). These results indicate that the differentially expressed circRNAs play a key role in testicular development, spermatogenesis, and sperm maturation.

### 3.5. Validation of circRNA Sequencing Results

**Figure 7:**
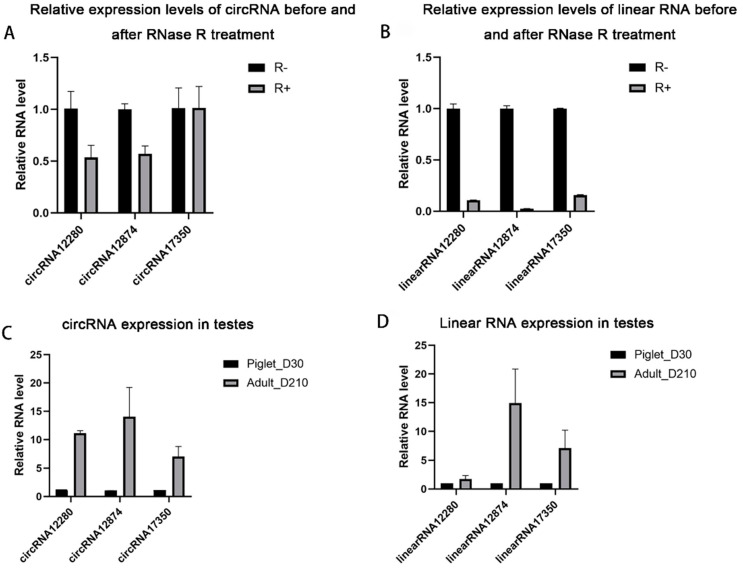
Validation of differentially expressed circRNAs in young (Piglet-D30) and sexual mature (Adult-D210) boar testes. Relative expression levels of circRNAs before and after RNase R treatment (**A**). Changes of the relative expression levels of linear RNA corresponding to circRNAs before and after RNase R treatment (**B**). Relative expression changes of circRNAs in testes before and after sexual maturity (**C**). Changes of the relative expression levels of linear RNA corresponding to circRNAs in the testes before and after sexual maturity. R− and R+ represent RNA treat with or without RNase R. The data were normalized against endogenous housekeeping gene *EF1α1* and the Value for the blank control was set as one (**D**).

To validate the reliability of the circRNA sequencing data, three differentially expressed circRNAs including circRNA 12280, circRNA 17350, and circRNA 12874 were selected for abundance analysis by RT-qPCR. First, RNase R treatment showed that these candidate circRNAs are resistant to RNase, suggesting their circular characteristics (Figure 7A,B). Second, RT-qPCR was used to detect their expressions at different developmental stages. The expression patterns of these selected circRNAs were found to be consistent with those obtained from the circRNA sequencing data (Figure 7C), confirming that the results obtained by circRNA sequencing are reliable.

## 4. Discussion

The testicle is the organ where male animals produce androgens and sperm and is the main organ to maintain male fertility. The fertility of boars is an extremely important indicator in production and genetic breeding. The time required for development to sexual maturity of different breeds of pigs is not consistent. Our results of H & E staining showed that seminiferous tubules in the testis tissues of sexually mature Landrace boars were actively involved in spermatogenesis, demonstrating that the 210-day-old Landrace boars exhibit physiological characteristics of sexual maturity.

In the present study, most of the circRNAs were 100–10,000 nt in length, which is slightly different from previous observations on human testes and seminal plasma samples [25]. This means different species may have different requirements for the length of circRNAs transferred from nucleus to cytoplasm. Most of the circRNAs were from exon regions, which is in line with the findings of Chen [40], who reported that exon regions and 5′UTR sequences produce the most circRNAs.

Some studies have shown that circRNA plays an important regulatory role in testicular development [41]. For example, a large number of circRNAs were identified in the testicular tissue or seminal plasma of Qinchuan cattle [24], rats [28], humans [25,42], and pigs [26]. Related research on pigs has been mainly focused on maps of the testicular transcriptome [43], miRNAs [44], and sperm [45]. However, the comparative expression and the potential function of circRNA in development of pig testis have not been hitherto investigated. Liang et al. profiled expression landscape of circRNAs in testis of pigs at a certain age instead of providing differently expressed circRNAs. Previously, it was demonstrated that the characteristics of circRNAs in pig testis are similar but not identical to those from other tissues. Compared with other organs, circRNA expression in pig testes is abundant, second only to that in the brain [26]. Such a result is consistent with the results for different mouse tissues [28], which may be due to the need for large-scale and accurate regulation of gene spatiotemporal expression during spermatogenesis.

In this study, 2326 significantly differently expressed circRNAs were found between the Piglet-D30 and Adult-D210 samples. It has been previously reported that some exon circRNAs serve as sponges for miRNAs, targeting source genes in the cytoplasm [20] and adsorbing these miRNAs to regulate miRNA-targeted genes. Some EIciRNAs have been found to be cis regulators of source genes in the nucleus, and circRNAs have also recently been found to be active participants in gene regulation [22].

It was previously reported that the productions of circRNAs and piRNAs in mouse spermatogenic cells were highly regulated instead of random processes [46]. Testicular circRNA was suggested related to a switch of the cellular process to overcome a particular challenge that may arise in the early stages of steroid production; in some cases, isoforms and circular transcripts from different genes share functions and a global regulation of circRNA production is established [47]. In our GO enrichment and KEGG signal pathway analyses of the source genes of differentially expressed circRNAs, it was found that these genes are mainly involved in biological processes such as the piRNA metabolic process, spermatogenesis, positive regulation of hormone biosynthetic process, cilium assembly, and germ cell development. This includes centrosome, motile cilium, cell-cell junction, sex chromatin, and other cellular components, as well as molecular functions such as phospholipid-translocating ATPase activity, transcriptional activator activity, and phosphatase binding, indicating that circRNAs are involved in spermatogenesis, sperm motility, meiotic cell cycle, and fertilization.

Previous study showed that SMARCA5-derived circRNA9244 could be used as a marker to determine the stages of testicular development [47]. Plasma estradiol levels were significantly lower in circRNA9244-higly expressed animals than those in circRNA9244 deficiency animals, which means that immature animals have low abundance of circRNA9244. Our sequencing results also showed that circRNA 10187, sourced from the *SMARCA5* gene was significantly downregulated in the sexual maturation group (Table S4). Androgen is essential for maintaining normal testicular spermatogenesis. Testis is not only the site of androgen biosynthesis, but also the target organ of androgen [48]. Our sequencing results also showed that, compared with the immature group, circRNA 6682 (derived from *NSD1* gene) involved in the androgen receptor binding is significantly upregulated in the testis of the sexually mature group (Table S4). NSD1 is homology to putative histone lysine methyl transferase that is observed in the testes of mangrove rivulus fish [49]. It is generally believed that androgen cannot directly act on spermatogenic cells, but on Sertoli cells and myoid cells to create a microenvironment suitable for spermatogenesis [50]. Our sequencing results showed that, some circRNAs involved in the spermatogenesis (e.g., circRNA 10979, derived from *POC1A* gene), germ cell development (e.g., circRNA 18456, derived from *TDRD1* gene) are significantly upregulated in the testis of the sexually mature group (Tables S4 and S5), which indicates that the testis of this group was more active. POC1A is essential for normal function of both Sertoli cells and germ cells [51]. Tudor domain-containing proteins (TDRDs) play a critical role in germ cell development and piRNA biogenesis [52]. These circRNAs might be involved in spermatogenesis by interacting with source genes. Due to the stability and spatiotemporal specificity of circRNAs, circRNA 10187, circRNA 6682, circRNA 10979, and circRNA 18456 may be used as biomarkers of boar sexual maturity. The study of these genes and the circRNAs produced by these genes may provide a new perspective for spermatogenesis research.

Meanwhile, the tight junction between Sertoli cells is an important structure of the blood testis barrier, which plays a key role in spermatogenesis [53]. Our sequencing results showed that the differentially expressed circRNAs were significantly enriched in tight junction pathway. For example, circRNA 1774 (derived from *CDC42* gene) and circRNA 18184 (derived from *PTEN* gene) were significantly downregulated in the testis of the sexually mature group (Table S6). The possible explanation is that after the blood testis barrier is established and matured, spermatogenic cells in the testis undergo meiosis, and a large number of spermatogenic cells increase continuously, which makes the proportion of Sertoli cells decrease with testis development [54]. Therefore, in different developmental stages of boar testes, some cell junction associated proteins may be reduced due to the lower proportion of Sertoli cells during testicular development. Our sequencing results also showed that the differentially expressed circRNAs were significantly enriched in mammalian target of rapamycin (mTOR) complex pathway. For example, circRNA 40370 (derived from *RICTOR* gene) is significantly upregulated in the testis of the sexually mature group (Table S6). This may be because of the importance of RICTOR/mTOR complex 2 signaling in spermatogenesis and Sertoli cell function through maintenance of BTB integrity, Sertoli cell cytoskeletal dynamics, and cell polarity [55].

## 5. Conclusions

The results indicated that circRNAs are abundantly and dynamically expressed in porcine testicular development and circRNAs may have important functions in porcine testicular development. Thus, this study can provide potential molecular markers used to evaluate the testicular developmental status of boars and selecting optimal breeding stock.

## Data Availability

The datasets generated for this study can be found in NCBI GEO accession GSE166000.

## Supplementary Materials

The following are available online at https://www.mdpi.com/article/10.3390/ani11051430/s1, Table S1: Summary of sequencing results in testis, Table S2: Testis-specific primer sequences used in this study, Table S3: List of all circRNAs expressed in testis, Table S4: Summary of differentially expressed circRNAs in testicular tissues of young and sexually mature Landrace boars, Table S5: GO analysis of host genes producing DECs in testicular tissues of young and sexually mature Landrace boars, Table S6: KEGG analysis of host genes producing DECs in testicular tissues of young and sexually mature Landrace boars.
